# Whole-body magnetic resonance for staging and response assessment of lymphoma in a pregnant woman treated with antenatal chemotherapy

**DOI:** 10.1259/bjrcr.20150293

**Published:** 2016-10-07

**Authors:** Domenico Albano, Caterina Patti, Donatella Narese, Antonino Mulè, Massimo Midiri, Massimo Galia

**Affiliations:** ^1^Department of Radiology, DIBIMED, University of Palermo, Palermo, Italy; ^2^Department of Hematology I, Azienda Ospedali Riuniti Villa Sofia-Cervello, Palermo, Italy

## Abstract

A 32-year-old pregnant female presented with bilateral supraclavicular swelling, diffuse itching and right shoulder pain. After lymph nodal biopsy, a diagnosis of nodular sclerosis Hodgkin’s lymphoma was obtained. A multidisciplinary team decided to start chemotherapy before the delivery, and whole-body MRI was used to stage the disease and evaluate the response after antenatal chemotherapy. This case shows that whole-body MRI is an attractive procedure that avoids radiation exposure and contrast administration, and enables staging and follow-up of a pregnant patient without risk to the fetus.

## Case presentation

A 32-year-old female at pregnancy week 22 was admitted to the haematology department with bilateral supraclavicular swelling, diffuse itching and right shoulder pain. Ultrasound imaging showed pathological bilateral supraclavicular enlarged lymph nodes, which were biopsied. A diagnosis of nodular sclerosis Hodgkin’s lymphoma (HL) was made.

Fetal ultrasound screening was normal. In view of the patient’s desire to proceed with the pregnancy, a multidisciplinary follow-up (by haematologists, gynaecologists, radiologists and neonatologists) was scheduled.

Combined fludeoxyglucose positron emission tomography (FDG-PET)/CT scan was not performed before the delivery because of the risk of radiation exposure during pregnancy.

**Figure 1.**
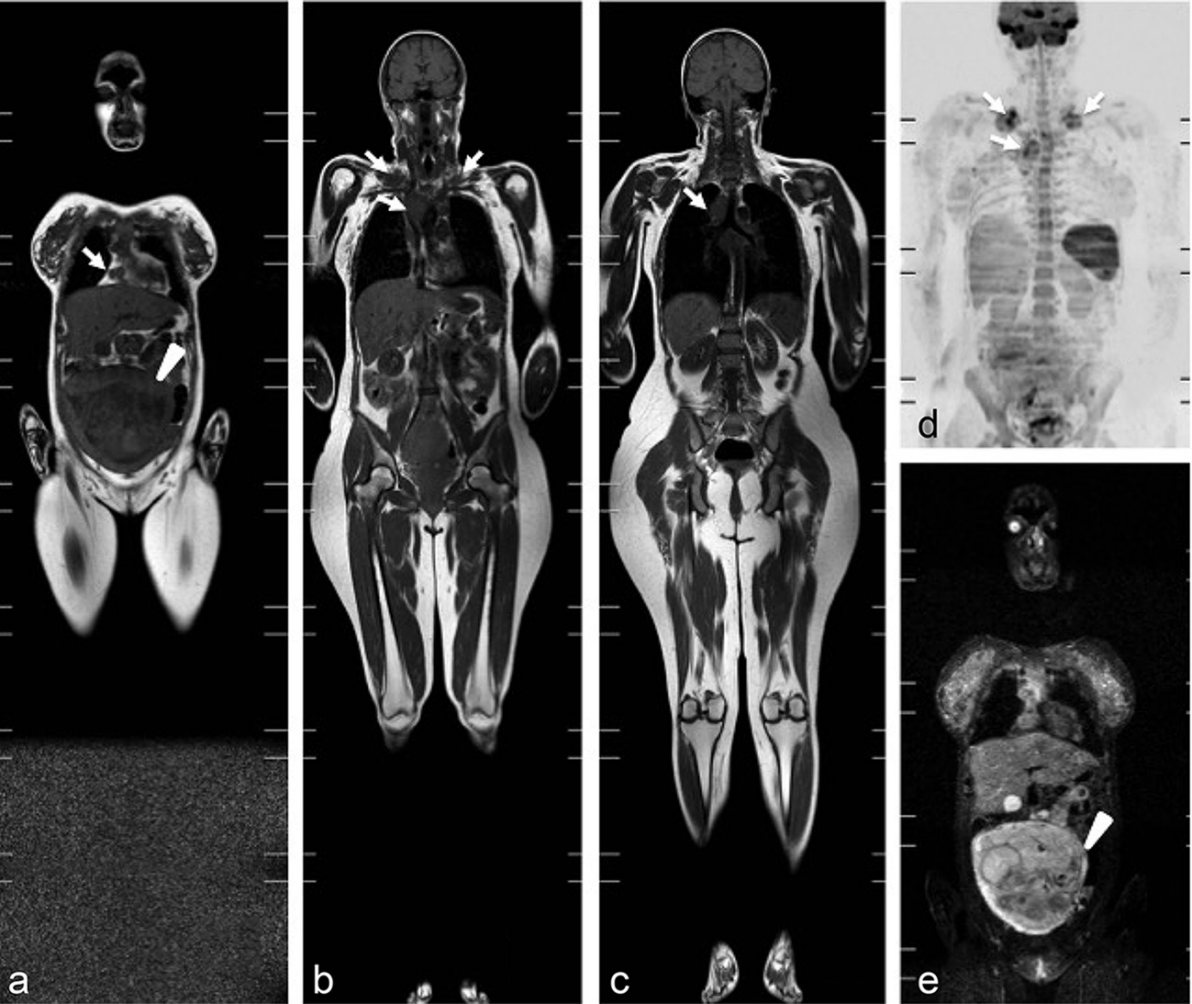
A 32-year-old pregnant female with Hodgkin’s lymphoma. Pregnancy week 23 (a, e; arrowheads). Coronal *T*_1_ weighted (a–c), coronal maximum intensity projection diffusion-weighted (d) and coronal short tau inversion-recovery (e) whole-body MRI showing bilateral supraclavicular, cardiophrenic and mediastinal lymph node involvement (a–d; arrows).

It was decided to stage the disease through ultrasound and whole-body MRI (WB-MRI), which showed right cervical, bilateral supraclavicular and mediastinal lymph node involvement without a bulky mass (axial diameter 4.7 × 4.1 cm), indicating Stage IIA disease, according to Ann Arbor classification^1^ (Figure 1).

The multidisciplinary team decided to start chemotherapy only if clinically necessary. Accordingly, the patient was followed up with ultrasound, WB-MRI, haematology consults and gynaecological examinations. Furthermore, therapy with steroid and paracetamol was started to slow down the progression of disease, and treat the itching and shoulder pain.

At pregnancy week 27, WB-MRI demonstrated a slight progression of the disease, seen as an enlargement of the lymph node packages, the largest was in the mediastinum with an axial diameter of 5.4  ×  4.5 cm.

At pregnancy week 29, the clinical conditions got worse and the patient presented with coughing, night sweating, numbness and pain radiating across the shoulder down the arm. An ultrasound examination showed enlargement of a right axillary lymph node and the internal jugular vein thrombosis that was treated with enoxaparin, which allowed complete recanalization in just a week. Thus, it was decided to start chemotherapy.

**Figure 2.**
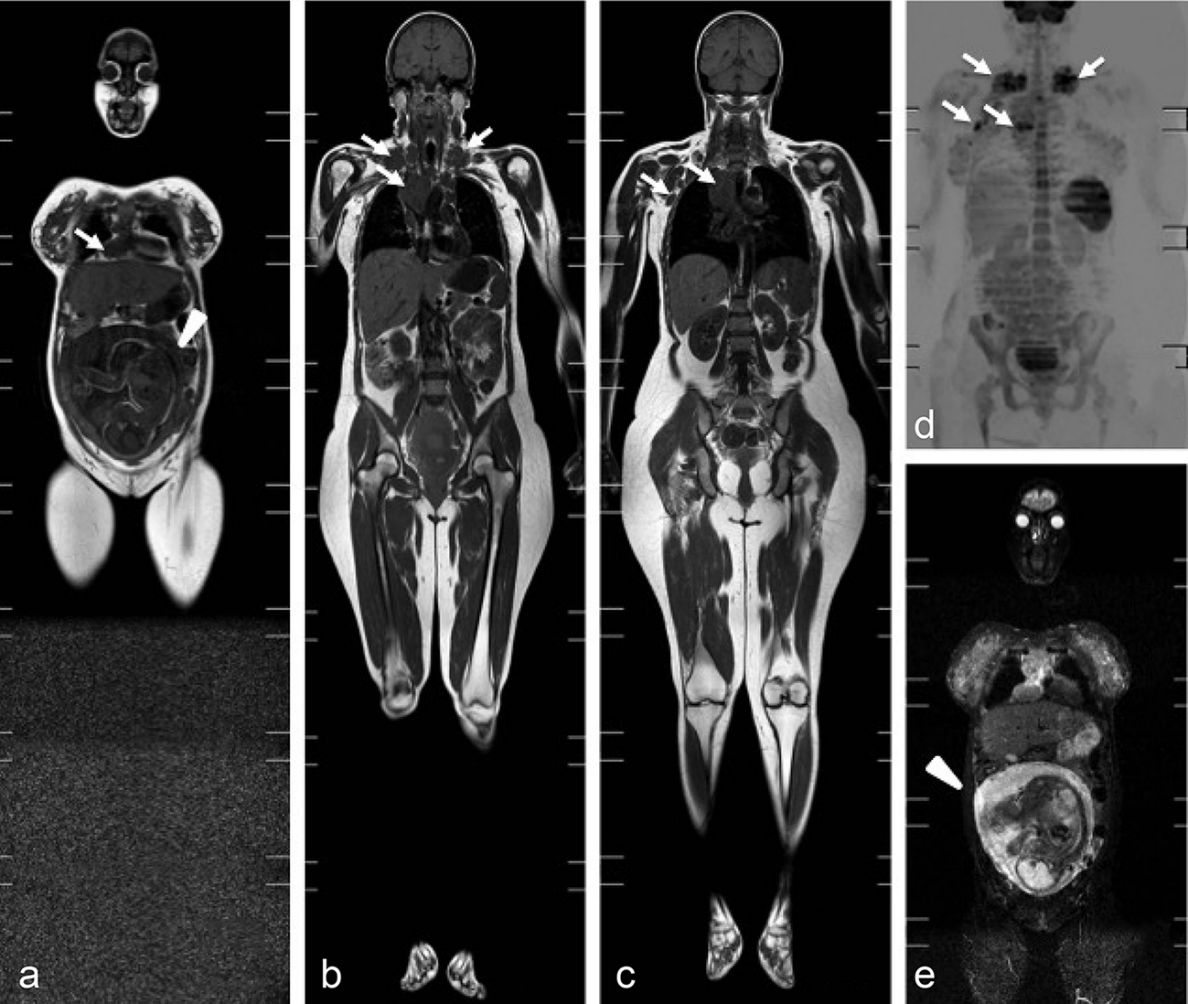
Pregnancy week 30 (a, e; arrowheads). Coronal *T*_1_ weighted (a–c), coronal maximum intensity projection diffusion-weighted (d) and coronal short tau inversion-recovery (e) whole-body MRI showing progression of the disease with right axillary involvement (c; arrows) and further enlargement of other lymph nodes involved (a–d; arrows).

WB-MRI was performed at pregnancy week 30, before the beginning of chemotherapy, confirming the progression of disease with right axillary involvement and further enlargement of the supraclavicular and mediastinal lymph nodes (the largest lesions with axial diameters of 5.8 × 4.7  cm), but without the involvement of extranodal or subdiaphragmatic sites; so the disease stage had not changed (Figure 2).

The patient received two courses of chemotherapy (adriamycin, bleomycin, vinblastine and dacarbazine) and showed an improvement in her clinical status a few days after the beginning of therapy, with disappearance of the coughing and sweating, and a decrease in the cervical swelling, numbness and pain.

**Figure 3.**
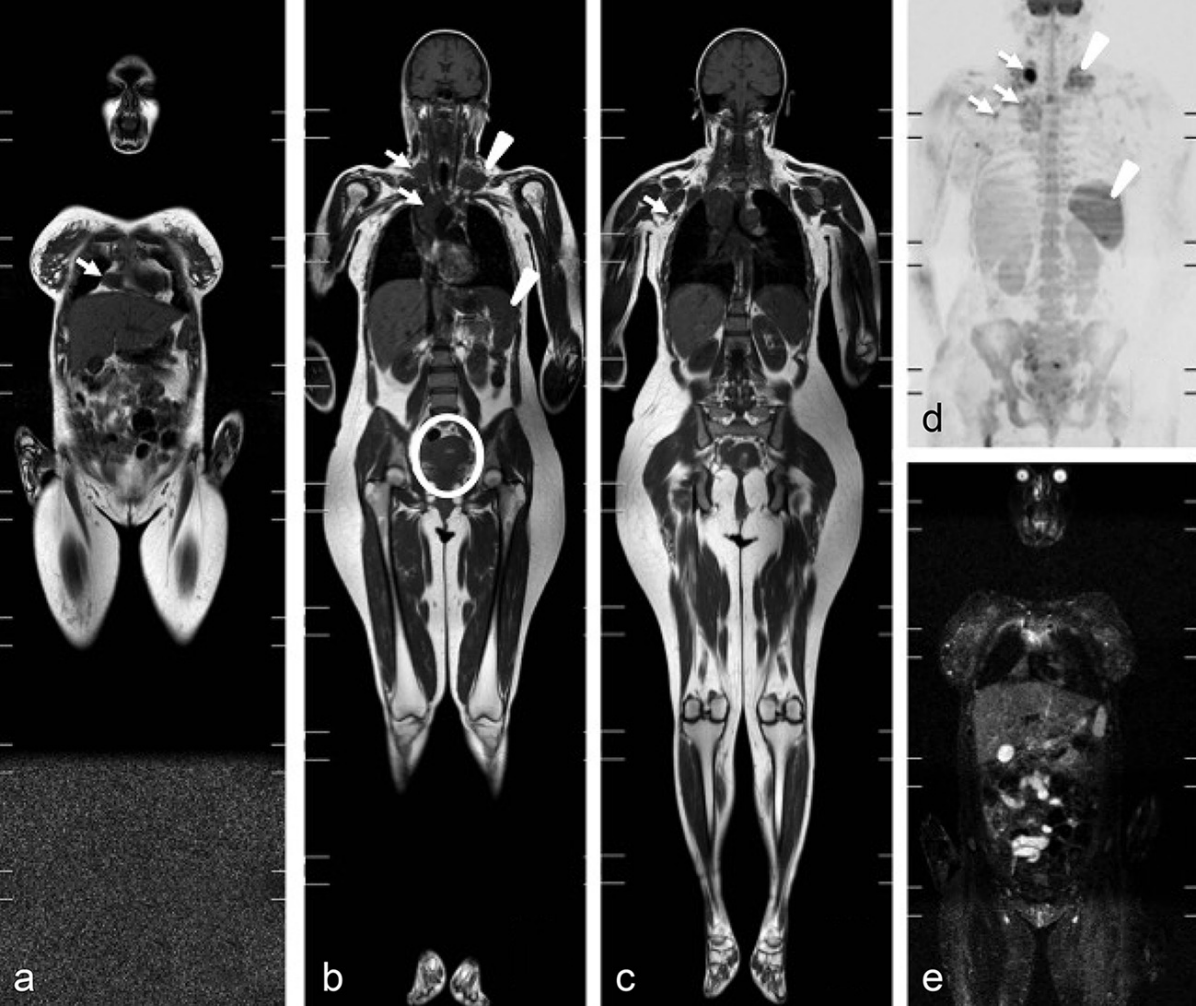
Images from scanning performed 2 weeks after delivery. Coronal *T*_1_ weighted (a–c), coronal maximum intensity projection diffusion-weighted (d) and coronal short tau inversion-recovery (e) whole-body MRI showing dimensional decrease of the axillary, cardiophrenic, right supraclavicular and mediastinal lymph nodes (a–d; arrows), a slight enlargement of the spleen and of left supraclavicular lymphadenopathy (b, d; arrowheads). Note also a still enlarged uterus after delivery (b; circle).

At pregnancy week 38, the patient gave birth to a healthy child, with a weight of 3.110 kg, *via* spontaneous delivery. A week after delivery, the patient presented with worsening of clinical symptoms, the reappearance of sweating and asthenia, signs of increased cervical swelling, and she received another course of adriamycin, bleomycin, vinblastine and dacarbazine. Ultrasound and WB-MRI were then performed to assess the response to treatment and showed progression of the supraclavicular lymphadenopathy and an enlargement of the longest axial diameter of the spleen from 11 to 14.5 cm, concluding that the disease was a resistant lymphoma (Figure 3).

**Figure 4.**
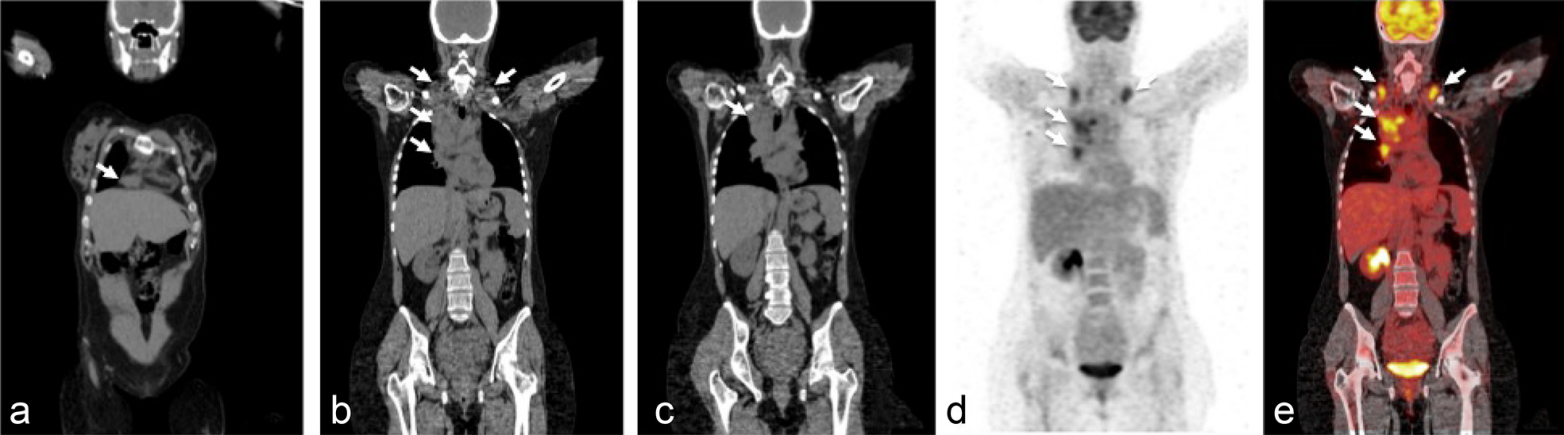
Images from scanning performed 2 weeks after delivery. Fludeoxyglucose-positron emission tomography/CT images (a–e) showing multiple areas of increased uptake in cardiophrenic, bilateral supraclavicular and mediastinal lymph nodes (a–e; arrows).

After WB-MRI, the patient underwent FDG-PET/CT scan that confirmed the MRI findings (Figure 4).

After confirming the findings, the haematologists decided to change the chemotherapy regimen and thus the patient received three courses of escalated bleomycin, etoposide, doxorubicin, cyclophosphamide, vincristine, procarbazine and prednisone and three standard bleomycin, etoposide, doxorubicin, cyclophosphamide, vincristine, procarbazine and prednisone.

**Figure 5.**
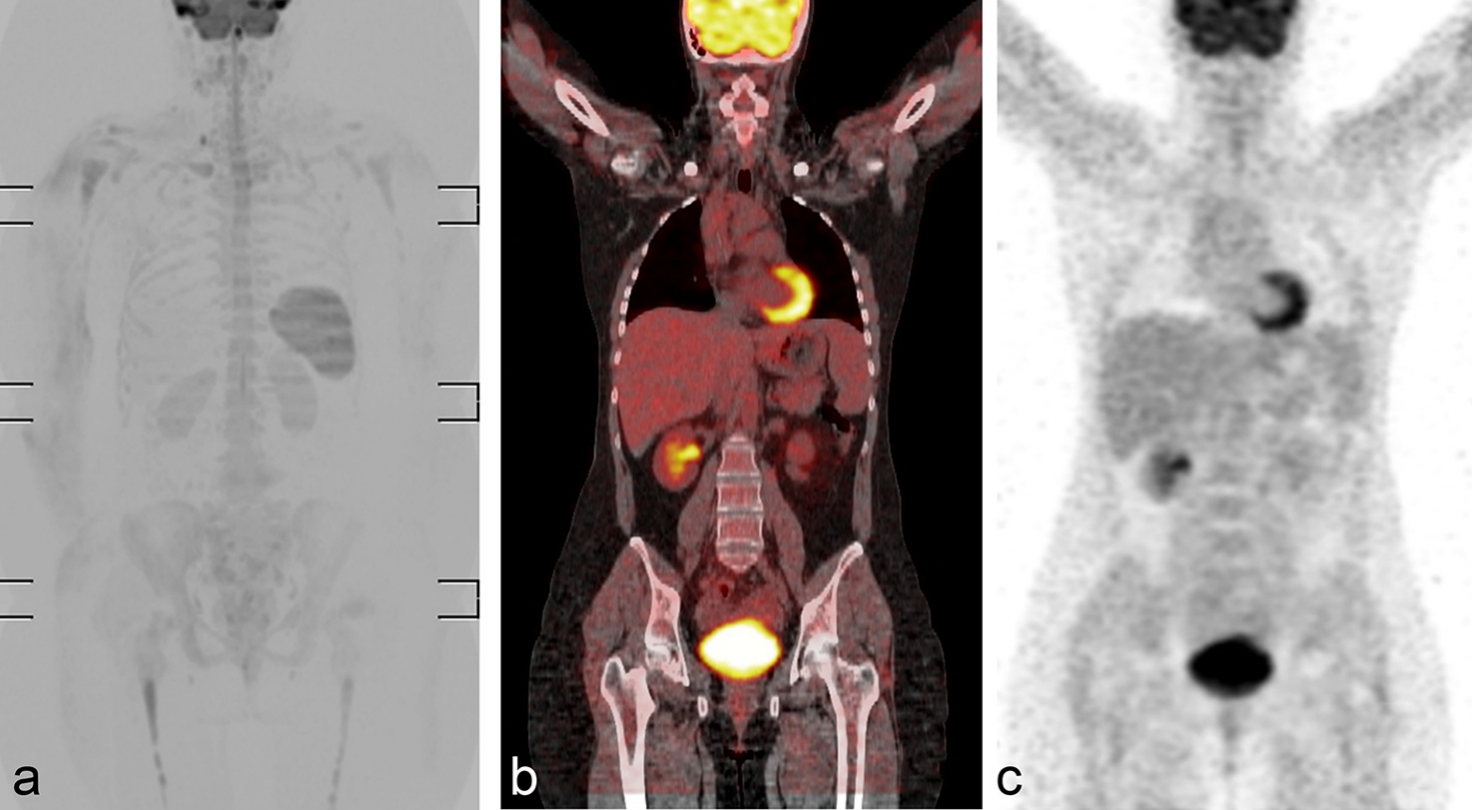
Images from scanning performed 1 month after the end of bleomycin, etoposide, doxorubicin, cyclophosphamide, vincristine, procarbazine and prednisone regimen. Coronal maximum intensity projection diffusion-weighted (a), fludeoxyglucose- positron emission tomography/CT (b) and fludeoxyglucose-positron emission tomography (c) images showing complete remission of the disease.

After chemotherapy, the patient underwent WB-MRI and FDG-PET/CT, which showed disappearance of the nodal locations of the disease and normal splenic size (Figure 5).

## Discussion

Lymphoma is the fourth most frequent cancer in pregnancy. HL is more common than non-HL and has an estimated prevalence of 1 per 6000 pregnancies.^2^ However, there are few published reports^3–9^ regarding lymphoma-specific data during pregnancy, concerning diagnosis, management, optimal timing of therapy, complications and outcomes when chemotherapy is started during gestation.

Only a small number of cases have demonstrated that antenatal chemotherapy with standard regimens (non-antimetabolite) during the second or third trimester does not increase morbidity or mortality for the fetus.^3–5^

The choice of starting chemotherapy during gestation depends on the presenting clinical signs and symptoms, gestational age at diagnosis and fetal risks with regard to the risk of antenatal chemotherapy against the potential adverse effect of delaying curative therapy. A multidisciplinary team of experts is required, as in our case, to develop an individualized management plan.

Accurate staging of newly diagnosed lymphoma is crucial to plan appropriate treatment. The goal of radiological staging is to provide guidance about the disease to help evaluate if treatment can be deferred to after delivery or whether the patient requires antenatal chemotherapy.

FDG-PET/CT are the reference standard to stage and evaluate response to treatment of many neoplasms,^10^ including several malignancies that can occur during pregnancy such as lymphoma; however, both techniques are contraindicated in pregnancy owing to their teratogenic risk.^11,12^ There are no large studies on fetal radiation exposure from FDG-PET/CT, and the levels of radiation exposure does not seem to be so high;^12^ however, the choice of performing FDG-PET/CT during pregnancy needs careful evaluation of the benefits and risks and the protocol should be modified to minimize fetal exposure. As FDG-PET/CT is strongly recommended in HL, our haematologists decided to perform FDG-PET/CT after the delivery to be sure there were no other sites of the disease not identified by WB-MRI.^1^

WB-MRI is a well-established radiation-free technique with a proven reliability in the staging and follow-up of lymphoma.^13–15^ WB-MRI and ultrasound may be appropriate techniques for staging and following up HL during pregnancy after the third month of gestation, which has not been associated with adverse fetal effects^11^.

Our WB-MRI protocol included a coronal *T*_1_ weighted turbo spin-echo sequence [repetition time (TR)/echo time (TE), 322/18 ms; slice thickness/gap 6/1 mm; craniocaudal coverage, 185.5 cm], a half-Fourier multishot *T*_2_ weighted inversion recovery turbo spin-echo sequence (TR/TE/inversion time 1498/64/165 ms; slice thickness/gap 6/1 mm; cranio-caudal coverage, 185.5 cm) and an axial diffusion-weighted WB-MRI with background body signal suppression sequence (b values of 0 and 800 s/mm^2^; TR/TE, 3134/64 ms; slice thickness/gap, 6/0 mm; craniocaudal coverage, 96 cm). A built-in body receiver coil was used. Images were acquired under free breathing, except for the chest and abdomen, which were acquired using breath-holding. Mean WB-MRI examination time was 30–35 min, including patient positioning. Our relatively fast protocol makes WB-MRI scan a quite acceptable examination. Furthermore, the conjunction between morphological and functional imaging sequences is helpful in order to interpret MRI findings.^16^

In conclusion, the presented case shows that WB-MRI is an attractive procedure that avoids radiation exposure and contrast administration, and enables staging and following up a pregnant patient with lymphoma without risk to the fetus.

## Learning points

1. Pregnant patients with lymphoma can be treated with chemotherapy but the choice to start therapies during gestation requires a multidisciplinary team of experts.
2. Accurate staging of newly diagnosed lymphoma is crucial to plan appropriate treatment but FDG-PET/CT scan is contraindicated in pregnancy owing to its teratogenic risk.
3. WB-MRI avoids radiation exposure or contrast administration and enables staging and following up a pregnant patient without fetal risk.

## Consent

Written informed consent for the case to be published (including images, case history and data) was obtained from the patient for publication of this case report.
